# Postmating–prezygotic isolation between two allopatric populations of *Drosophila montana*: fertilisation success differs under sperm competition

**DOI:** 10.1002/ece3.1995

**Published:** 2016-02-16

**Authors:** Outi Ala‐Honkola, Michael G. Ritchie, Paris Veltsos

**Affiliations:** ^1^ Department of Biological and Environmental ScienceUniversity of JyvaskylaPO Box 35FI‐ 40014JyvaskylaFinland; ^2^ Centre for Biological DiversitySchool of BiologyUniversity of St AndrewsSt AndrewsKY16 9TSUK; ^3^ Department of Ecology and EvolutionUniversity of LausanneBiophore BuildingLausanne1015Switzerland

**Keywords:** Ejaculate tailoring, ejaculate–ejaculate interaction, postcopulatory sexual selection, reproductive isolation, speciation

## Abstract

Postmating but prezygotic (PMPZ) interactions are increasingly recognized as a potentially important early‐stage barrier in the evolution of reproductive isolation. A recent study described a potential example between populations of the same species: single matings between *Drosophila montana* populations resulted in differential fertilisation success because of the inability of sperm from one population (Vancouver) to penetrate the eggs of the other population (Colorado). As the natural mating system of *D. montana* is polyandrous (females remate rapidly), we set up double matings of all possible crosses between the same populations to test whether competitive effects between ejaculates influence this PMPZ isolation. We measured premating isolation in no‐choice tests, female fecundity, fertility and egg‐to‐adult viability after single and double matings as well as second‐male paternity success (P_2_). Surprisingly, we found no PMPZ reproductive isolation between the two populations under a competitive setting, indicating no difficulty of sperm from Vancouver males to fertilize Colorado eggs after double matings. While there were subtle differences in how P_2_ changed over time, suggesting that Vancouver males’ sperm are somewhat less competitive in a first‐male role within Colorado females, these effects did not translate into differences in overall P_2_. Fertilisation success can thus differ dramatically between competitive and noncompetitive conditions, perhaps because the males that mate second produce higher quality ejaculates in response to sperm competition. We suggest that unlike in more divergent species comparisons, where sperm competition typically increases reproductive isolation, ejaculate tailoring can reduce the potential for PMPZ isolation when recently diverged populations interbreed.

## Background

Reproductive isolation is traditionally classified into premating and postmating isolation, which have been extensively studied (Coyne and Orr 2004). More recently postmating–prezygotic (PMPZ – occurring after ejaculation but before fertilisation) isolation has been recognized as important (Coyne and Orr 2004; Howard et al. 2009). The relative significance of these mechanisms to the process of speciation is a major question in speciation research (Butlin et al. 2012). Typically, PMPZ isolation in animals has been measured between species pairs (Metz et al. 1994; Shaw et al. 1994; Price et al. 2001; Matute and Coyne 2010; Manier et al. 2013b; Sweigart 2010; Sagga and Civetta 2011; Ahmed‐Braimah and McAllister 2012), while studies between populations of one species are more rare (Alipaz et al. 2001; Brown and Eady 2001; Fricke and Arnqvist 2004; Nosil and Crespi 2006; Jennings et al. 2011, 2014; Firman and Simmons 2014), even though these are more relevant to the initiation of reproductive barriers.

PMPZ isolation mechanisms operate at the level of gametic and/or reproductive protein interactions. They may involve sperm motility (Gregory and Howard 1994), sperm storage (Price et al. 2001), differential female use of stored sperm (Manier et al. 2013b), interactions between seminal fluids of competing males (Fry and Wilkinson 2004), and the inability of sperm to fertilise eggs (Jennings et al. 2014). Analogous processes can occur during pollen interactions with the stigma and style of plants (Howard 1999; Baack et al. 2015). Females of most animals mate with multiple males (Birkhead and Møller 1998) so that sperm from different males are usually in competition. This may influence PMPZ isolation mechanisms, and the interaction between sperm competition and PMPZ is potentially complex (Bella et al. 1992).

Sperm competition studies involving males of different species usually reveal conspecific sperm precedence (CSP), where homospecific sperm have a fertilisation advantage, even when single matings show no reproductive isolation (reviewed in Howard et al. (2009)). Coyne and Orr (2004) have called CSP one of the most significant findings in studies of reproductive isolation. The rapid evolution of reproductive proteins (e.g., Wyckoff et al. (2000), Swanson et al. (2001)), be it due to male–female coevolution, male–male competition, or sexual conflict (see the discussion in Pitnick et al. (2009)), has been suggested to drive incompatibilities between allopatric populations and can ultimately lead to speciation (Coyne and Orr 2004; Howard et al. 2009).

Sperm competition studies between populations within a species have revealed all possible outcomes: There is conpopulation sperm precedence in the cowpea seed beetle (*Callosobruchus maculatus*; Brown and Eady (2001)), guppies (*Poecilia reticulata*; Ludlow and Magurran (2006)), stalk‐eyed flies (*Teleopsis dalmanni*; Rose et al. (2014)) and two subspecies of *Drosophila pseudoobscura* (Dixon et al. 2003), while heteropopulation sperm precedence was found in yellow dung flies (*Scathophaga stercoraria*; Hosken et al. (2002)). In a study separating first‐ and second‐male effects on sperm competition success, Long et al. (2006) found that conpopulation males are more successful in the 2nd male role while heteropopulation males are more successful in the 1st male role in *Drosophila melanogaster*. In addition, no differences in fertilisation success between con‐ and heteropopulation males were found between two populations of house mouse (*Mus domesticus*; Firman and Simmons (2014)) and in crosses between two races (Dixon et al. 2003) and eight replicate populations of *D. melanogaster* (Arbuthnott et al. 2014). These varying outcomes suggest that complex factors affect male–female coevolution between populations (Rowe and Day 2006). They include the population divergence time (Coyne and Orr 2004), and the mechanisms behind sperm competition and interactions between sperm and egg.

In a noncompetitive, single mating setting, a strong postmating–prezygotic (PMPZ) isolation mechanism was recently described from crosses between two populations of *Drosophila montana* (Jennings et al. 2014). Direct observation under a dissecting microscope revealed that when Colorado females mated with Vancouver males, sperm reached the female sperm storage organs but very few eggs were fertilised, compared to the reciprocal cross or within‐population crosses. The PMPZ isolation involved the inability of sperm from Vancouver males to successfully penetrate and fertilise the eggs of Colorado females. Because *D. montana* females typically remate quickly (40% of females remate 4 h after the first mating (Aspi 1992)), understanding the significance of female multiple mating to the potential isolation is crucial, for example, when estimating the effects on reproductive isolation in a possible secondary contact of these populations in nature. In some *Drosophila* species, mating with a heterospecific male can have long‐lasting negative fitness consequences to the female even if the second mating is with her own species. For example, there is decreased egg production in such crosses between *D. santomea* and *D. yakuba* (Matute and Coyne 2010) and *D. americana* sperm interferes with *D. novamexicana* female reproduction leading to decreased offspring production (Ahmed‐Braimah and McAllister 2012). On the other hand, within species, the ejaculate of one male has the potential to increase or decrease the fertilisation success of a competitor (Simmons and Beveridge 2011; Locatello et al. 2013) and even affect the quality of the competitor's offspring through seminal fluid effects (Garcia‐Gonzalez and Simmons 2007; Crean et al. 2014). Also, *Drosophila* males are known to strategically tailor their ejaculates (both sperm numbers and seminal fluid composition) in competitive situations (Wigby et al. 2009; Lüpold et al. 2011; Sirot et al. 2011; Manier et al. 2013a), which means that ejaculate composition in competitive situations may be quite different from noncompetitive situations. Our aim here was to explore possible ejaculate × ejaculate interactions with female reproduction and male sperm competition success in these divergent *D. montana* populations.

To investigate the effects of multiple mating on reproductive isolation between Colorado and Vancouver populations of *D. montana,* we measured (1) premating isolation in no‐choice tests between the two populations (measured as mating latency, a typical measure of male attractiveness in *Drosophila* (Barth et al. 1997; Ritchie et al. 1999; Ala‐Honkola et al. 2013)), (2) female fecundity and fertility, and egg‐to‐adult viability of the offspring after single and double matings in order to detect possible heteropopulation ejaculate and ejaculate × ejaculate interaction effects on female reproduction, and (3) PMPZ isolation as sperm competition success of the 2nd male to mate (P_2_). We created double‐mated females in all possible combinations between these populations and measured P_2_ over 6 days in order to detect possible differences in sperm storage/fertilisation success of con‐ versus heteropopulation males (*D. montana* females tend to run out of sperm after 6 days (Aspi 1992)). We find no PMPZ isolation between these populations, in contrast to the results from the noncompetitive situation, and discuss potential reasons for the difference in outcomes.

## Methods

### Flies

**Figure 1:**
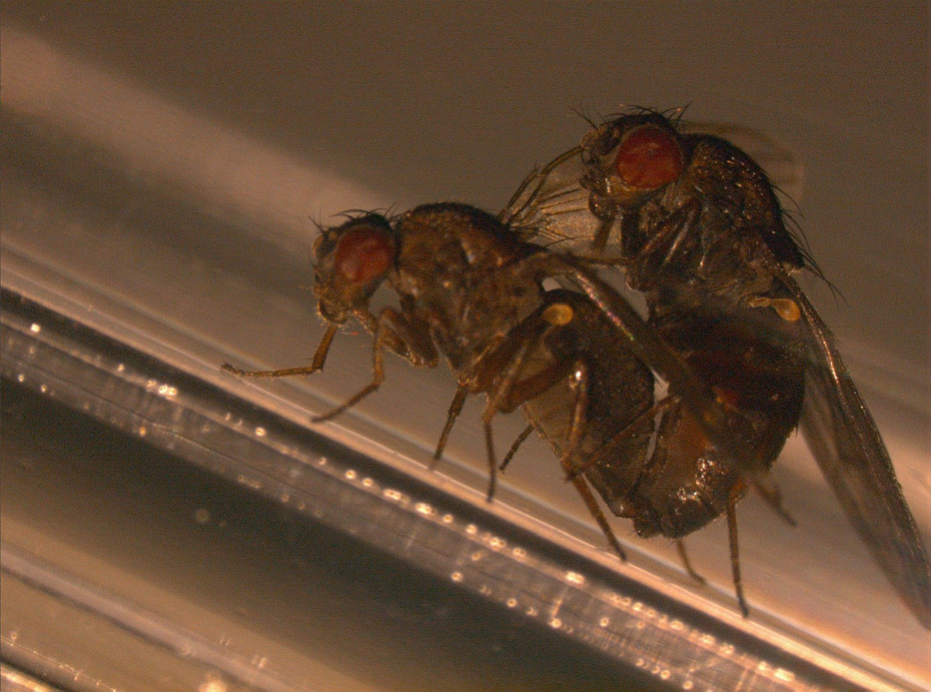
*Drosophila montana* mating pair.

Adult *D. montana* (see Fig. 1) were collected from riparian habitats in Vancouver (Canada) in 2008 and in Colorado (USA) in 2009. Once in the laboratory, isofemale lines were established for each wild‐caught female in half‐pint bottles on Lakovaara malt medium (Lakovaara 1969) until a large number of F3s were available. From each isofemale line (*N* = 20 for Vancouver, *N* = 13 for Colorado), 20 F3 males and 20 F3 females were then combined in a 25 × 25 × 60 cm wooden population cage with a Plexiglas top and eight available food bottles for feeding, oviposition, and larval rearing and bred in overlapping generations in constant light and temperature (18°C). Constant light is necessary to prevent flies from undergoing reproductive diapause (Lumme 1978). Virgin flies were collected from food bottles within three days of eclosion under CO_2_ anesthesia, kept in single sex vials (10 flies per vial) and used after maturation (which takes about 3 weeks) at the age of 26–30 days.

### Experimental setup

We performed all possible crosses between Colorado and Vancouver females and males to have a fully factorial design using 50 females per cross type (total *N* = 400). Over 4 days of mating trials, females were mated and remated on consecutive days, first the Colorado and then the Vancouver females. Hence, all females remated approximately 24 h after the initial mating. We recorded the time when flies were paired in a vial, as well as the beginning and end of copulation. After copulation, males were stored in 70% ethanol at −20°C until DNA extraction. Females were transferred to new vials before remating in order to count the eggs and offspring produced after the first mating. This allowed us to confirm that the first mating was successful and to measure female fecundity and fertility as well as egg‐to‐adult viability of the eggs laid after the first mating. Between 64% to 90% of females (depending on the cross) remated at our chosen remating time, so there was still variation in females’ propensity to mate at this time point. After the second mating, females were transferred to new vials twice at 2‐day intervals and all the eggs and eclosing offspring were counted from these vials. This allowed us to measure female fecundity and fertility as well as egg‐to‐adult viability of the eggs laid after remating and to detect possible time trends in the proportion of eggs sired by the 2nd male to mate (P_2_). After laying eggs for 6 days, females were stored in 70% ethanol at −20°C until DNA extraction.

### Paternity tests and population differentiation with SNP markers

For SNP genotyping, we randomly chose up to 15 offspring per time point (3 time points from days 1–2, 3–4, and 5–6) per female that produced at least 7 offspring per time point and had also produced offspring before the 2nd mating (i.e., the 1st mating was successful); in total, over 7000 offspring from 161 females (*N*(CCC) = 22, *N*(CVC) = 21, *N*(CCV) = 13, *N*(CVV) = 15, *N*(VVV) = 19, *N*(VCV) = 29, *N*(VVC) = 19, *N*(VCC) = 23; crosses are abbreviated by female population, 1st male, and 2nd male population, respectively: C, Colorado; V, Vancouver). DNA was extracted from whole flies using standard methods by KBiosciences (Herts, UK). SNP genotyping was performed with a PCR‐based KASP™ genotyping assay by KBiosciences (Herts, UK).

We used a subset of the genetic markers described in Veltsos et al. (2015), see Additional file 1 (Dryad Digital Repository: http://dx.doi.org/10.5061/dryad.085vq). The SNP markers were analyzed in Cervus v3.0.7 (Kalinowski et al. 2007). For paternity analyses, we typed 12 markers both in the offspring and in parents. Only the markers in Hardy–Weinberg equilibrium were used (10 retained, null allele frequency was <0.01 for all). For parentage analysis, a simulation was run in Cervus with simulated offspring set to 10,000, proportion of candidate parents sampled 1, minimum number of typed loci 8, and the remaining parameters at the default settings. Paternity analysis was then performed to identify the most likely father of each offspring with the following parameters (8 minimum typed loci, proportion sampled 1, proportion loci typed 0.99, proportion loci mistyped 0.01). The confidence level used was 0.99.

**Figure 2:**
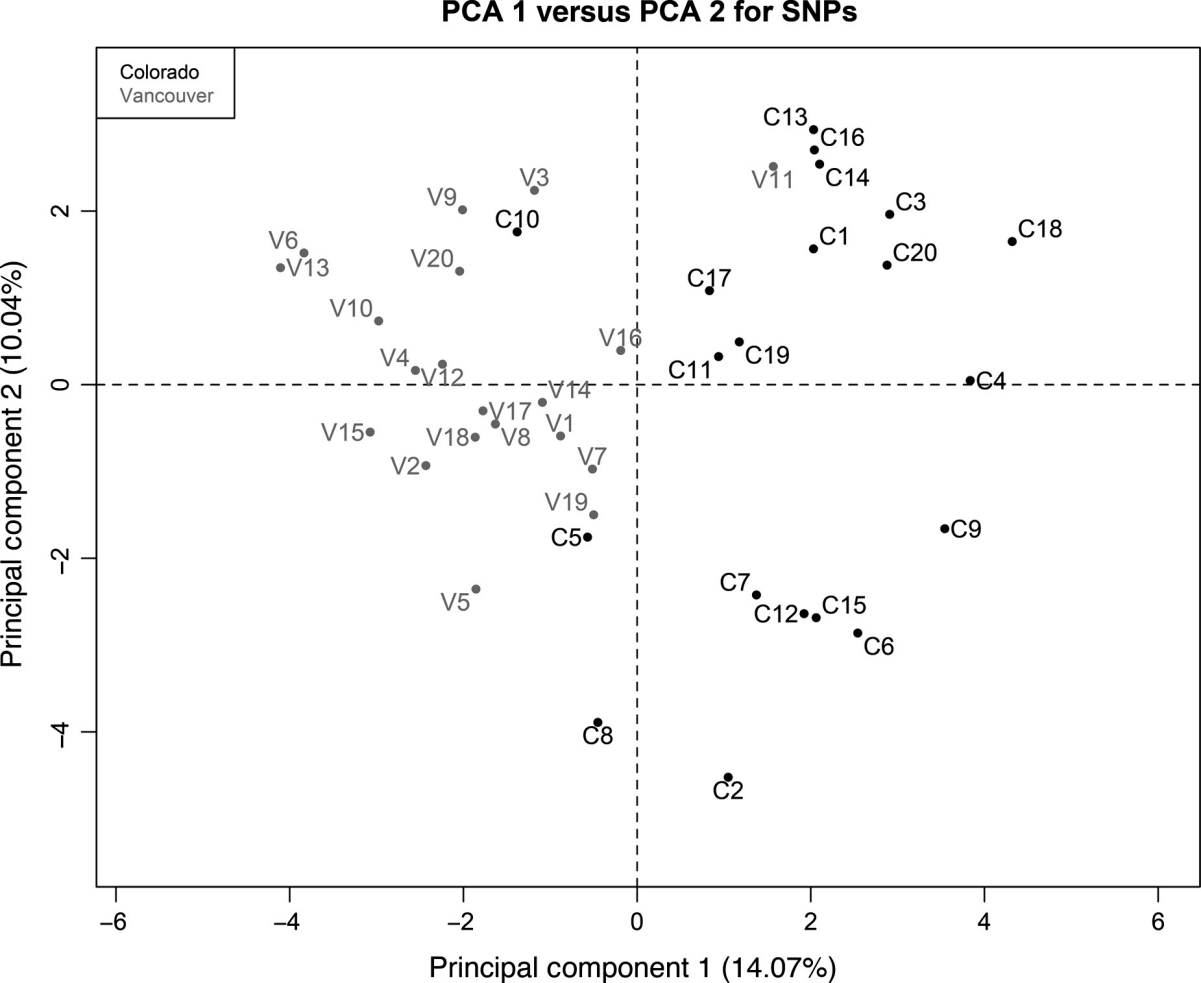
Principal component analysis conducted with 37 SNP markers. Each point represents an individual. Principal component 1 (14.07% of the variance) clearly differentiates the two populations.

To assess the genetic differentiation between parental populations, 20 individuals from each population cage were genotyped for 50 SNP markers, see Additional file 1 (Dryad Digital Repository: http://dx.doi.org/10.5061/dryad.085vq). The allele frequency analysis in Cervus was used to exclude markers that were not in Hardy–Weinberg equilibrium (or with F values >0.05 when the test was not carried out because the minimum allele frequency was not reached). The 37 remaining markers were analyzed by principal component analysis using the FactoMineR package (v 1.28) (Husson et al. 2015) in R version 3.1.1 (R Development core team, 2014), by converting the three possible allelic states (two homozygotes and heterozygote) to the numbers 1, 2, 3, with the heterozygote having the intermediate value when all three allelic states were observed. Principal component 1, which explained 14.07% of the variance, clearly differentiates between the populations (Fig. 2). We estimated the mean Fst to be 0.079, using the same markers, with the web version of Genepop (v 4.2) (Raymond and Rousset 1995; Rousset 2008). We also tested the significance of this observed Fst against simulated panmixis of the populations using a custom Python script that generated pairs of populations by randomly subsampling all parents to create two populations and calculating an Fst score of the subsamples. The mean simulated Fst after 10,000 iterations was 0.013 and its range was completely nonoverlapping with the observed Fst, providing strong support that the original individuals originated from significantly differentiated populations.

### Statistical analysis

We used R (version 3.1.3) for statistical analyses (R Development core team, 2015). To detect differences in proportions (for example, the proportion of mating versus nonmating and remating versus not remating flies) among treatments, we used a chi‐square test.

When analyzing traits measured after the first mating, we fitted female population, 1st male population and their interaction as factors into the full models. Mating latency (log_10_‐transformed) and copulation duration were analyzed with general linear models, number of eggs and offspring produced were analyzed with generalized linear models (GLM) with negative binomial distribution and logarithmic link function (function “glm.nb” in “MASS” package in R (Venables and Ripley 2002)), and egg‐to‐adult viability was analyzed with a GLM with quasibinomial error distribution (binomial model was overdispersed) and a logit link function with sample sizes as weights (function “glm”).

The number of eggs produced on the day after the first mating was analyzed after excluding cases with zero eggs to exclude infertile females and potential unsuccessful copulations. Egg‐to‐adult viability of the eggs laid after the first mating was analyzed with zero viabilities included (this could result from both unsuccessful copulations and cases of complete incompatibility) and without zeros to exclude all unsuccessful copulations. The number of progeny produced after the first mating was analyzed without zeros to exclude all unsuccessful copulations and complete incompatibilities that are included in viability measurements.

When analyzing traits measured after the second mating, we fitted female population, 1st male population, 2nd male population, and their interactions as factors. Remating latency (log_10_‐transfomed), 2nd copulation duration, and egg production after remating were analyzed with general linear models, progeny production after remating was analyzed with a GLM with negative binomial distribution, and egg‐to‐adult viability after remating and P_2_ were analyzed with a GLM with quasibinomial error distribution and a logit link function with sample sizes as weights. We excluded zeros from the analysis of egg and offspring production and egg‐to‐adult viability of the eggs laid after the second mating to exclude cases of unsuccessful copulations. From the analysis of P_2_, we excluded cases with P_2_ = 0 (*N* = 6) as those potentially represent an unsuccessful 2nd copulation (this species shows 2nd male sperm precedence with P_2_ typically around 0.6 to 0.7 (Aspi 1992; Ala‐Honkola et al. 2014)).

We analyzed time trends in P_2_ over our three time points (days after remating 1–2, 3–4 and 5–6) separately for both female populations in order to investigate retainment of heteropopulation sperm in female sperm storage organs (if 2nd male sperm is lost from storage faster than in the control treatment (CCC or VVV), we expect P_2_ to decrease over time, and if 1st male sperm is lost from storage, we expect P_2_ to increase over time compared to the control treatment). We therefore fitted cross type, time (as a continuous covariate), and their interaction as fixed effects into a generalized linear mixed model (GLMM) with female as a random factor (3 observations per female) using binomial error distribution (binomial model was not overdispersed) and a logit link function with sample sizes as weights (function “glmer” in library “lme4” (Bates et al. 2014a,b)). Total P_2_ over 6 days was also analyzed for both female populations separately with cross type as a factor (GLM with quasibinomial error distribution and a logit link function with sample sizes as weights).

Nonsignificant interactions were removed from the statistical models using backward selection. If significant interactions complicated the interpretation of the result, the data were analyzed separately for each female population. Statistical significance of factors and interactions was assessed with F‐test for general linear models (type III sums of squares) and for GLMs by comparing nested models with and without the factor with likelihood ratio test (LRT) or analysis of deviance test for quasibinomial distribution (F‐test result reported).

## **Results**

### Premating isolation of the first mating

During the first mating, there was no indication of premating isolation between populations in terms of mating probability. Colorado females mated equally likely with Colorado males (76 of 96) and Vancouver males (86 of 98) (*χ* ^2^ = 2.43, df = 1, *P* = 0.12). Similarly, Vancouver females mated equally likely with Colorado males (86 of 98) and Vancouver males (89 of 97) (*χ* ^2^ = 0.47, df = 1, *P* = 0.49). Also, there was no female population × 1st male population interaction in mating latency (*F*_1,333_ = 1.1, *P* = 0.30) that would be expected if some crosses were mating more slowly than others. In addition, neither female (*F*_1,334_ = 0.2, *P* = 0.62) nor male (*F*_1,334_ = 0.002, *P* = 0.96) population had an effect on mating latency (Table 1). Equally, there was no female population × 1st male population interaction in copulation duration (*F*_1,329_ = 2.1, *P* = 0.15), but copulations of Vancouver females were on average 13 s shorter than those of Colorado females (*F*_1,330_ = 4.5, *P* = 0.034) and not affected by male population (*F*_1,330_ = 0.04, *P* = 0.84).

**Table 1:** Means, SDs, and sample sizes for traits measured for the first mating in each cross. The female population is referred first in each cross: C, Colorado, V, Vancouver

Trait	Cross (F × M)			
	CC	CV	VC	VV
Mating latency (min)	68.2 (62.5), 76	61.6 (55.5), 86	63.4 (59.8), 86	71.3 (75.4), 89
Copulation duration (s)	260 (60.4), 76	268 (49.7), 84	256 (58.5), 86	246 (52.2), 87
Number of eggs produced before remating (zeros excluded)	22.6 (11.2), 65	21.4 (10.4), 82	25.3 (12.9), 80	18.5 (11.9), 69
Egg‐to‐adult viability (zeros included)	0.39 (0.27), 65	0.37 (0.32), 82	0.61 (0.29), 80	0.65 (0.33), 69
Egg‐to‐adult viability (zeros excluded)	0.46 (0.22), 54	0.47 (0.28), 64	0.64 (0.26), 76	0.73 (0.25), 61
Number of progeny produced before remating (zeros excluded)	10.6 (6.0), 54	11.0 (7.3), 64	15.8 (9.1), 76	13.3 (7.2), 61

### Postmating isolation following the first mating

The probability of not laying any eggs in the 24 h following the first mating was actually higher in within‐ than between‐population crosses (CC: 10/75 females did not lay eggs, CV: 3/85; *χ* ^2^ = 3.90, df = 1, *P* = 0.048; VV: 20/89 females did not lay eggs, VC: 5/85; *χ* ^2^ = 8.42, df = 1, *P* = 0.004) suggesting that unsuccessful copulations, complete incompatibility of mating partners or cryptic female choice against close relatives were more likely within populations than between populations. The number of eggs produced (zeros excluded) after the 1st mating was not affected by female population (LRT = 0.06, df = 1, *P* = 0.80), although almost showed a significant female × male population interaction (LRT = 3.5, df = 1, *P* = 0.06). Females mated with Vancouver males produced fewer eggs than those mated with Colorado males (LRT = 7.2, df = 1. *P* = 0.007, Table 1).

There was no indication of between‐population postmating incompatibility in the crosses after the 1st mating (female population × male population interaction in egg‐to‐adult viability was not significant: *F*_1,292_ = 0.1, *P* = 0.73), but Colorado females produced fewer viable eggs than Vancouver females (*F*_1,293_ = 36.1, *P* < 0.001, Table 1). Male population did not affect egg‐to‐adult viability of eggs laid after the first mating (*F*_1,293_ = 0.5, *P* = 0.47, Table 1). The result was similar when the pairs with zero egg‐to‐adult viability were excluded from the data (female × male population interaction: *F*_1,251_ = 0.3, *P* = 0.60; female population: *F*_1,252_ = 31.8, *P* < 0.001; male population: *F*_1,252_ = 1.8, *P* = 0.18, Table 1). Excluding these removed both unsuccessful copulations and cases of completely incompatible mating pairs.

**Table 2:** The number of females that remated and did not remate in each cross. Crosses are abbreviated by female population, 1st male, and 2nd male population, respectively: C, Colorado; V, Vancouver

Cross (*F* × M_1 _× M_2_)	CCC	CVC	CCV	CVV	VVV	VCV	VVC	VCC
Remated	33	36	25	28	38	31	32	35
Did not remate	4	6	14	14	4	9	13	7
% remating	89%	86%	64%	67%	90%	78%	71%	83%

Progeny production after the 1st mating was not affected by a female × male population interaction (LRT = 1.9, df = 1, *P* = 0.18). Vancouver females produced more offspring (LRT = 13.7, df = 1. *P* < 0.001, Table 1) than Colorado females, despite laying fewer eggs as their egg‐to‐adult viability was higher. Male population had no effect on offspring production (LRT = 0.06, df = 1. *P* = 0.80).

### Premating isolation of the second mating

Remating probabilities of Colorado females differed among crosses (*χ* ^2^ = 10.8, df = 3, *P*‐value = 0.013; Table 2) because they were less likely to remate with Vancouver than Colorado males (*χ* ^2 ^= 9.4, df = 1, *P*‐value = 0.002; Table 2). Vancouver females, on the other hand, remated equally likely in all crosses (*χ* ^2^ = 5.6, df = 3, *P*‐value = 0.13, Table 2).

Colorado males were more attractive 2nd males than Vancouver males, because their remating latency was shorter than that of Vancouver males (*F*_1,189_ = 14.4, *P* < 0.001, Table 3). Vancouver females remated sooner than Colorado females (*F*_1,189_ = 3.9, *P* = 0.050, Table 3), and 1st male population had no effect on remating latency (*F*_1,189_ = 0.9, *P* = 0.34).

**Table 3:** Mean (SD) and sample size for traits measured after remating for each cross. Crosses are abbreviated by female population, 1st male, and 2nd male population, respectively: C, Colorado; V, Vancouver

Trait	Cross (F × M_1_ × M_2_)							
	CCC	CVC	CCV	CVV	VVV	VCV	VVC	VCC
Remating latency (min)	72 (57), 27	58 (57), 27	107 (74), 16	97 (55), 19	70 (62), 24	99 (72), 29	62 (67), 21	65 (74), 30
2nd copulation duration (s)	283 (58), 27	273 (47), 27	274 (107), 16	276 (53), 19	247 (52), 24	289 (66), 29	283 (50), 21	240 (59), 30
Number of eggs produced after remating (zeros excluded)	155 (44), 27	151 (47), 26	158 (60), 15	145 (55), 18	149 (38), 22	168 (35), 28	162 (46), 20	168 (39), 26
Number of progeny produced after remating (zeros excluded)	97 (40), 27	112 (41), 26	104 (46),15	110 (42), 18	112 (36), 22	115 (27), 28	100 (29), 20	118 (36), 26
Egg‐to‐adult viability after remating (zeros excluded)	0.60 (0.16), 27	0.73 (0.12), 26	0.68 (0.19), 15	0.77 (0.18), 18	0.76 (0.18), 22	0.69 (0.15), 28	0.64 (0.13), 20	0.70 (0.14), 26
P_2_	0.58 (0.24), 21	0.66 (0.15), 16	0.61 (0.25), 12	0.62 (0.18), 13	0.67 (0.19), 16	0.65 (0.19), 27	0.73 (0.19), 18	0.70 (0.18), 22

In the analysis of 2nd copulation duration, the three‐way female × 1st male × 2nd male population interaction was significant (*F*_1,185_ = 7.2, *P* = 0.008) and therefore these data were analyzed separately for the two female populations. For Colorado females, the population of the 1st (*F*_1,86_ = 0.12, *P* = 0.73) or the 2nd male (*F*_1,86_ = 0.04, *P* = 0.85) did not affect 2nd copulation duration, but for Vancouver females, 2nd copulation duration depended on the combination of 1st and 2nd male population (Table 3: 1st male population × 2nd male population interaction: *F*_1,100_ = 7.2, *P* = 0.008).

### Postmating isolation following the second mating

The crosses did not differ in female fecundity or fertility, as the number of eggs produced after remating was not affected by female population (*F*_1,178_ = 2.3, *P* = 0.13), 1st male population (*F*_1,178_ = 2.4, *P* = 0.12) or 2nd male population (*F*_1,178_ = 0.3, *P* = 0.61; Table 3). Similarly, the number of offspring produced (i.e., fertility) after remating was not affected by female population (LRT = 0.7, df = 1, *P* = 0.39), 1st male population (LRT = 0.2, df = 1, *P* = 0.67) or 2nd male population (LRT = 0.008, df = 1, *P* = 0.93; Table 3).

**Figure 3:**
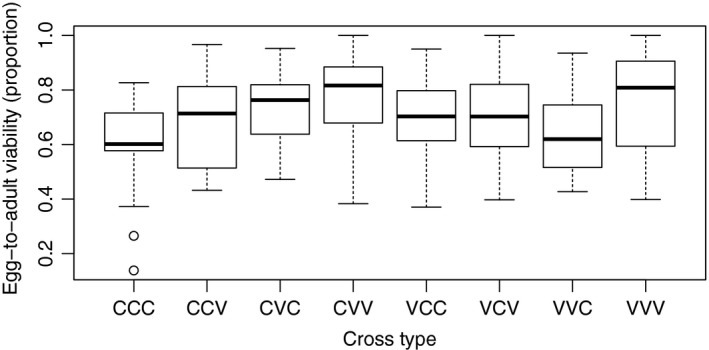
Egg‐to‐adult viability after remating in the eight crosses performed. Crosses are abbreviated by female population, 1st male, and 2nd male population, respectively. C, Colorado; V, Vancouver.

Egg‐to‐adult viability of the eggs laid after remating was dependent on the combination of the female population and the 1st male population (female population × 1st male population interaction significant: *F*_1,177_ = 6.2, *P* = 0.014), and therefore, the data were analyzed separately for the two female populations. Colorado females that first mated with Colorado males had a lower egg‐to‐adult viability after remating than those first mated to Vancouver males (*F*_1,83_ = 11.3, *P* = 0.001; Table 3, Fig. 3), while the 2nd male population had no effect on egg‐to‐adult viability (*F*_1,83_ = 0.4, *P* = 0.54; Table 3). For Vancouver females, egg‐to‐adult viability depended on the combination of the 1st male and the 2nd male population (1st male population × 2nd male population interaction significant: *F*_1,92_ = 4.6, *P* = 0.034). Egg‐to‐adult viability was the highest when both males were from Vancouver, but the lowest when a Vancouver male was followed by a Colorado male (see Fig. 3, Tables 3 and 4).

**Table 4:** Final model (GLMs with quasibinomial error distribution) of factors explaining variance in Vancouver females’ egg‐to‐adult viability after remating

Effect	Parameter estimate	SE	*t*‐value	*P*
Intercept (Colorado 1st & 2nd male)	0.86	0.15	5.9	<0.001
1st male Vancouver	−0.37	0.22	−1.7	0.087
2nd male Vancouver	−0.09	0.20	−0.5	0.644
Vancouver 1st and 2nd male	0.69	0.31	2.2	0.029

For P_2_ analysis, we had offspring paternity data for 145 females and on average 33.0 (SD 9.1, range 7–45) offspring per female (4785 in total) were assigned to a sire with 99% confidence. The total P_2_ over six days was not affected by female population (*F*_1,141_ = 2.6, *P* = 0.11), 1st male population (*F*_1,141_ = 1.5, *P* = 0.23) or 2nd male population (*F*_1,141_ = 0.03, *P* = 0.85; Table 4).

**Figure 4:**
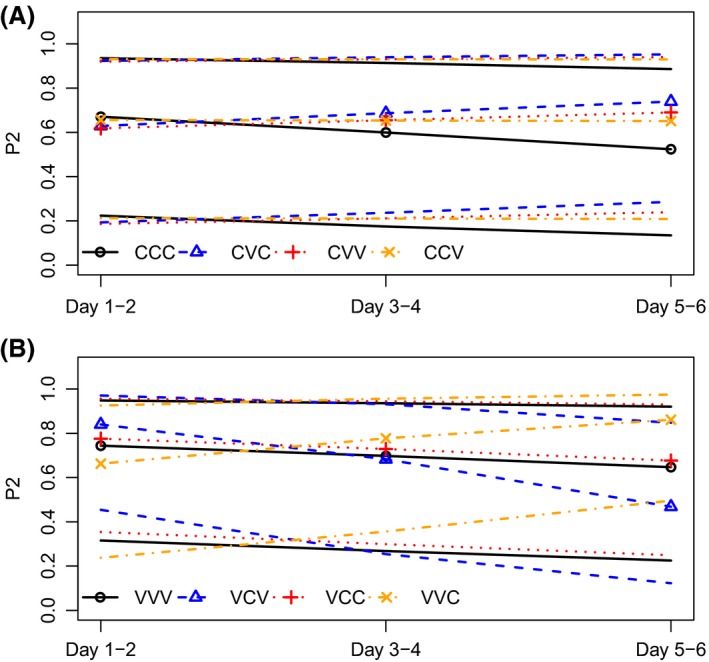
Model predictions and 95% confidence intervals from GLMM for P_2_ over time in different crosses for Colorado (A) and Vancouver (B) females. Crosses are abbreviated by female population, 1st male, and 2nd male population, respectively. C, Colorado, V, Vancouver.

For both female populations, P_2_ changed differently over time among crosses (see Fig. 4 for model predictions and 95% confidence intervals for each cross and Table 5 for the statistical model), that is, the cross × time interaction was significant (Colorado: LRT = 14.3, df = 3, *P* = 0.002; Vancouver: LRT = 92.7, df = 3, *P* < 0.001). There was no indication that heteropopulation males would have a lower success in the 2nd male role compared to conpopulation males, because P_2_ did not decrease faster in the heteropopulation versus con‐population cross as expected if heteropopulation sperm was lost from storage faster than own population sperm. This is demonstrated by the similarity of time effects on P_2_ in the CCV compared to the CCC cross (*P* = 0.3; Fig. 4 and Table 5), and the fact that P_2_ increased instead of decreased over time in the VVC compared to the VVV cross (*P* < 0.001; Fig. 4 and Table 5). If heteropopulation males would have a lower success in the 1st male role compared to conpopulation males, P_2_ of conpopulation males would increase faster when heteropopulation males mate first, compared to when conpopulation males mate first. This pattern would suggest that heteropopulation sperm was lost from storage faster than conpopulation sperm. This is what we found for Colorado females: P_2_ increased over time in the CVC cross compared to CCC cross (*P* < 0.001; Fig. 4 and Table 5). However, we found the opposite effect for Vancouver females: P_2_ decreased faster over time in the VCV cross than in the VVV cross (*P* < 0.001; Fig. 4 and Table 5). This suggests that Colorado sperm are very competitive in matings with Vancouver females, regardless of mating order, while Vancouver sperm are not as competitive when mating in 1st male role with Colorado females. However, the total P_2_ over 6 days did not differ among crosses (Colorado: *F*_3,58_ = 0.7, *P* = 0.57; Vancouver: *F*_3,79_ = 0.5, *P* = 0.66).

**Table 5:** Final GLMMs (binomial distribution) of factors explaining variance in P_2_ in the two female populations. Pure crosses are set as intercepts and therefore *P*‐values refer to the difference between the respective factor and the intercept. *P* values for cross × time interactions have been Bonferroni corrected (i.e., multiplied by 3) to account for multiple comparisons (comparison of cross × time interaction of each cross to that of the control cross). Bolded *P*‐values indicate that the difference in time trend between the pure cross and the respective cross is statistically significant. Crosses are abbreviated by female population, 1st male, and 2nd male population, respectively: C, Colorado; V, Vancouver

Effect	Parameter estimate	SE	*z*‐value	*P*
Intercept (CCC cross)	1.01	0.33	3.1	0.002
CCV cross	−0.36	0.54	−0.7	0.50
CVC cross	−0.75	0.48	−1.6	0.12
CVV cross	−0.70	0.53	−1.3	0.19
time	−0.31	0.11	−2.8	0.005
CCV × time	0.30	0.18	1.6	0.30
CVC × time	0.57	0.16	3.6	**0.001**
CVV × time	0.47	0.18	2.6	**0.03**
Intercept (VVV cross)	1.30	0.36	3.6	<0.001
VCC cross	0.19	0.48	0.4	0.68
VCV cross	1.25	0.46	2.7	0.007
VVC cross	−1.20	0.49	−2.4	0.014
time	−0.23	0.12	−1.9	0.06
VCC × time	−0.02	0.16	−0.1	1
VCV × time	−0.66	0.16	−4.2	**<0.001**
VVC × time	0.81	0.17	4.7	**<0.001**

## Discussion

Single mating crosses previously found PMPZ reproductive isolation between Colorado females and Vancouver males, with sperm from Vancouver males showing low fertilisation success with eggs of Colorado females (Jennings et al. 2014). Here, we have tested for PMPZ isolation following double matings between the same populations, with the potential for more complex ejaculate–ejaculate and ejaculate–female interactions. Surprisingly, we did not find a major reduction of sperm competitiveness of Vancouver males in double matings. Our premating isolation results also differ from those of Jennings et al. (2014), but this likely reflects different experimental setups. Any postmating effects found here were subtle. We found evidence for male population effects (possibly ejaculate and ejaculate–ejaculate interaction effects) on female egg laying and egg‐to‐adult viability.

We did not find premating isolation between Colorado and Vancouver populations during females’ first mating. Neither the probability to mate nor mating latency differed among crosses. However, in the females’ second mating, there was asymmetric premating isolation between the populations, with Colorado females being less likely to remate with Vancouver males, and Vancouver females mating equally likely with males from both populations. The shorter remating latency of Colorado males suggests that they were more attractive than Vancouver males, which is consistent with their higher frequency courtship song (Klappert et al. 2007). High‐frequency courtship song is usually more attractive to females in this species (Aspi and Hoikkala 1995; Ritchie et al. 1998; Veltsos et al. 2012). Vancouver females had a shorter refractory period after mating as they accepted matings sooner than Colorado females. This fits with our remating data: Colorado females remated less, especially with the apparently less attractive Vancouver males. Remating latency was not affected by the first‐male population, suggesting that males from Vancouver and Colorado did not differ in their ability to delay female remating.

We failed to replicate the strong premating isolation detected in the same populations (Jennings et al. 2014). This difference is perhaps because of the different experimental setup. We used no‐choice tests as our main goal was to obtain double‐mated females for sperm competition analysis, whereas Jennings et al. (2014) used multiple choice tests. For example, it has been shown in crosses between *D. arizonae* and *D. mojavensis* that experimental design can dramatically affect behavioral isolation mechanisms: smaller mating chambers that likely allowed flies to compare several potential mates led to an increase in isolation (Jennings and Etges 2010), which reflects the natural situation between *D. arizonae* and *D. mojavensis*. A recent meta‐analysis also showed that female mating preferences are stronger in choice designs (Dougherty and Shuker 2015). For *D. montana*, a choice design is likely to reflect the natural mating system better than a no‐choice situation as several males have often been found to court one female in the field (Aspi et al. 1993).

We did not see the strong postmating reproductive isolation between Colorado females and Vancouver males that was found earlier for single matings (Jennings et al. 2014). In the 24 hr after the first mating, egg‐to‐adult viability was not affected by the female × male population interaction, though Colorado females had lower egg‐to‐adult viability (below 50%) than Vancouver females (above 60%), while male population had no effect. Low viability of eggs laid early is well known in *D. melanogaster* and is explained by inefficient fertilisation due to the release of mature eggs before sperm storage is complete (Chapman et al. 2001). Egg‐to‐adult viabilities after the second mating were indeed considerably higher than after the first mating (Tables 1 and 3).

Population effects on egg‐to‐adult viability after the 2nd mating were more complex than after the 1st mating due to the female population × 1st male population interaction, and these data were analyzed separately for the two female populations. Again, we found no indication that Vancouver males showed impaired fertilisation success with Colorado females (Jennings et al. 2014). Colorado females that first mated with Colorado males had a lower egg‐to‐adult viability after remating than those first mated to Vancouver males but 2nd male population had no effect on egg‐to‐adult viability. The persistence of a 1st male effect on egg‐to‐adult viability, even though 2nd males fertilised more eggs (P_2_ ≈ 0.6), was surprising, but not unique. It has been shown previously that males of the Australian field cricket (*Teleogryllus oceanicus*) with high embryo viability increase the viability of embryos sired by inferior males (Garcia‐Gonzalez and Simmons 2007). In our case, Colorado 1st males decreased the viability of 2nd males. More dramatic 1st male effects were recently reported in the fly *Telostylinus angusticollis*: 1st male phenotype (large size) was transferred to the offspring of the 2nd male through seminal fluid effects (Crean et al. 2014).

For Vancouver females, egg‐to‐adult viability depended on the combination of the male populations and was highest when both males were from Vancouver, but lowest when a Vancouver male was followed by a Colorado male, suggesting ejaculate × ejaculate interaction effects, with poorer performance of Vancouver males under a competitive situation. Such first‐male ejaculate effects and ejaculate × ejaculate interaction effects on egg‐to‐adult viability beyond the 2nd mating are likely to explain why offspring viabilities of males differ after single and double matings (Droge‐Young et al. 2012). Despite the effects on egg‐to‐adult viability, our crosses did not differ in egg and offspring production and thus mating with heteropopulation males did not have harmful effects on female reproduction as described between crosses of different species of *virilis* group flies (Sweigart 2010; Sagga and Civetta 2011; Ahmed‐Braimah and McAllister 2012).

Our paternity analysis further confirms that Vancouver males do not show a major decline in the ability to fertilise Colorado females after double matings. P_2_ measured over 6 days was not affected by female, 1st or 2nd male population or any interaction. However, there were subtle differences in how P_2_ changed over time among the crosses, with Vancouver males’ paternity success declining more quickly when in competition with Colorado sperm within Colorado females in the defensive (1st male) role. These effects did not translate into differences in overall P_2_ over 6 days, however. Colorado males, on the other hand, were very good in sperm defense (i.e., in 1st male role) within Vancouver females. Over both female populations, P_2_ did not decrease faster when heteropopulation males mated second compared to when conpopulation males mated second, which suggests that heteropopulation sperm was not lost from female sperm storage faster than conpopulation sperm.

A large reduction in fertilisation success of Vancouver sperm with Colorado eggs was recently described (Jennings et al. 2014). Why did we not find a similarly strong effect here? Interactions between ejaculates or strategic differences in ejaculate composition may influence the success of sperm under competitive conditions and could account for the difference between the two studies. However, we also did not see evidence of large effects in the 24 h after the first mating, before sperm competition could occur. Our results seem unlikely to be due to differences in sample size and our experiment was performed only a year after the previous study, with the same source of flies. This difference in reproductive isolation between Colorado and Vancouver populations also cannot be explained by contamination of the populations in the laboratory due to “stray” flies because we could clearly differentiate the populations using 37 SNP markers (Fig. 2). The most likely source of the difference is the time frame during which single mating effects were measured, which differs between the two studies. We measured single mating effects for one day only, while Jennings et al. (2014) measured them for up to 7 days. It is possible that the fertilisation success of Vancouver sperm decreases rapidly as sperm age (Snook and Hosken 2004; Radhakrishnan and Fedorka 2011) in the reproductive tract of Colorado females and our one‐day time frame was not long enough to detect this decreased fertilisation capacity of Vancouver sperm. Unfortunately, since completing this experiment, the Vancouver stock has been lost, preventing the replication of the Jennings et al. (2014) study design.

Our result, that Vancouver males do not show a major decline in the ability to fertilise Colorado females after double matings, could be explained by strategic ejaculate tailoring. In *Drosophila*, males in several species ejaculate more sperm to mated than virgin females (Lüpold et al. 2011; Manier et al. 2013a), because larger ejaculates displace more previously stored sperm and lead to higher paternity success of second males (Manier et al. 2010). In addition to sperm numbers, other aspects of ejaculates can be tailored; Australian field cricket males manipulate sperm viability by increasing viability in competitive situations (Thomas and Simmons 2007). It was further shown that seminal fluid can affect sperm viability and crickets that invest in high viability ejaculate can enhance the viability of rival sperm (Simmons and Beveridge 2011). Holman (2009) showed that in *D. melanogaster*, seminal fluid of one male can protect the sperm of another male. If similar mechanisms occur in *D. montana*, the high quality ejaculates that second males produce in response to sperm competition could explain why (first) male population has no effect on fertilisation success under sperm competition while it does after single mating (Jennings et al. 2014) (it is also notable that egg‐to‐adult viability is good when two Vancouver males mate with a Colorado female, Fig. 3). Second‐male ejaculate may thus enhance the fertilisation ability of first‐male sperm. Our understanding of seminal fluid tailoring in response to sperm competition is still at its infancy, but we already know that this occurs in *D. melanogaster* (Wigby et al. 2009; Sirot et al. 2011).

Vancouver and Colorado populations have recently diverged (during the last glaciation (Mirol et al. 2007)) and it is possible that in such young population pairs ejaculate tailoring can have striking positive effects on fertilisation capacity of rival sperm that are not seen in species pairs (e.g., Manier et al. 2013b), in which sperm competition typically increases reproductive isolation (Howard et al. 2009). Our results suggest that the previously reported PMPZ isolation in single matings (Jennings et al. 2014) would not lead to reproductive isolation if the populations came in contact in nature. Further studies comparing reproductive isolation in population pairs under single and multiple mating are needed in order to evaluate the generality of our finding.

## Conclusions

We found that the previously reported reproductive isolation in single matings between Colorado females and Vancouver males does not translate to differences in sperm competition success as assayed by double matings. A potential explanation is that males increase the competitive quality of their ejaculate in response to sperm competition, which enhances first‐male fertilisation ability and thus masks the reproductive isolation seen in a noncompetitive setting.

## Conflict of Interest

The authors declare that they have no competing interests.

## Availability of Supporting Data

Data and Additional file 1 available from the Dryad Digital Repository: http://dx.doi.org/10.5061/dryad.085vq.
